# Comparison of the effect of air tamponade versus no tamponade after pars plana vitrectomy for idiopathic epiretinal membrane

**DOI:** 10.1038/s41598-021-84442-z

**Published:** 2021-03-03

**Authors:** Kyuhwan Jang, Daniel Duck-Jin Hwang, Jayoung Ahn, Gisung Son, Ji In Park, Joonhong Sohn

**Affiliations:** 1 Department of Ophthalmology, Hangil Eye Hospital, 35 Bupyeong-daero, Bupyeong-gu, Incheon, 21388 South Korea; 2 Department of Ophthalmology, Catholic Kwandong University College of Medicine, Incheon, South Korea; 3 Department of Medicine, Kangwon National University Hospital, Kangwon National University School of Medicine, Chuncheon, Gangwon-do South Korea

**Keywords:** Diseases, Eye diseases, Retinal diseases

## Abstract

This study aimed to compare the surgical outcomes of pars plana vitrectomy (PPV) with and without air tamponade in patients with idiopathic epiretinal membrane (iERM). We prospectively enrolled 145 patients with iERM who underwent a 25-gauge transconjunctival sutureless PPV. Patients were assigned to either the air tamponade (air) group (79 eyes) or balanced salt solution (BSS; no tamponade) group (66 eyes). The central macular thickness (CMT), peripapillary retinal nerve fiber layer (pRNFL) thickness, and best-corrected visual acuity (BCVA) were compared for two years. At baseline, there were no significant differences between the two groups. CMT and BCVA were not significantly different between the groups for 2 years. However, the air group had a significantly lower thickness in the superior temporal pRNFL sector at 1 month (*p* = 0.01) and in the inferior temporal and superior temporal pRNFL sectors at 3 months (*p* = 0.02 for both). There were no significant differences between both groups in all the pRNFL sectors from 6 months to 2 years. The outcomes of PPV with air tamponade and that with no tamponade appear to be equivalent. This shows that air tamponade may not be an imperative procedure for iERM surgery and has no additional benefit.

## Introduction

Idiopathic epiretinal membrane (iERM), is a common cause of visual impairment in the elderly population^1,2^. As the fibrocellular contraction of iERM progresses, irregular wrinkling of the inner retinal layer occurs and, consequently, more severe full thickness retinal distortion can occur^3^. In patients with symptomatic visual disturbances, timely surgical removal of iERM provides restoration of the foveal structure and prevents visual disturbances^4,5^. Pars plana vitrectomy (PPV) and epiretinal membrane peeling have been widely accepted as a standard procedure for iERM in patients with visual symptoms^6,7^.

Multiple studies have been conducted on the effect of internal limiting membrane (ILM) peeling on the prognosis of iERM surgery^8–12^. Despite controversy^13^, ILM peeling has become more widely accepted because it is known to reduce the recurrence of iERM^8,11,14^. However, no clinical studies have assessed the long-term effects of intraocular tamponade during PPV for iERM.

Air, which is commonly used as tamponade material, stays in the intraocular space temporarily until it is replaced with tissue fluid. It provides a sufficient tamponade effect with less discomfort compared to the use of intraocular gas tamponade^15^. According to previous reports, visual field defects were observed in patients who underwent vitrectomy with air tamponade, which may be caused by the process of fluid air exchange^16,17^. However, another study showed that air tamponade results in faster anatomical recovery compared to no tamponade^18^.

Thus, our aim was to assess whether the addition of air tamponade affects postoperative clinical outcomes of standard PPV and ILM peeling as a surgical repair of iERM. We compared the change in peripapillary retinal nerve fiber layer (pRNFL) and macular thickness, as well as visual acuity after PPV.

## Results

A total of 145 eyes were included in the study, with 79 eyes in the air group and 66 eyes in the BSS group. At baseline, there was no significant difference in age, sex, BCVA, IOP, CMT, or global pRNFL thickness between the two groups (Table 1). In both groups, there was no case of postoperative hypotony, vitreous hemorrhag, or endophthalmitis. Additionally, there was no case of reoperation due to postoperative complications, such as retinal detachment and recurrence of ERM.

**Table 1:** Comparison of patient characteristics of all study eyes with iERM at baseline.

Parameter	Total (N = 145)	BSS (N = 66)	Air (N = 79)	*p* value
Age	65.27 ± 8.29	64.95 ± 8.65	65.53 ± 8.03	0.678*
Sex (male/female)	50/95	24/42	26/53	0.663^†^
Operated eye (right/left)	76/69	35/31	41/38	0.892^†^
Lens status (phakic/IOL)	132/13	63/3	69/10	0.090^†^
VA (logMAR)	0.35 ± 0.25	0.33 ± 0.25	0.36 ± 0.25	0.425*
AXL (mm)	23.48 ± 1.14	23.41 ± 0.86	23.54 ± 1.33	0.496*
SE (D)	− 0.19 ± 2.40	0.08 ± 1.90	− 0.43 ± 2.73	0.203*
IOP (mmHg)	15.24 ± 2.70	15.62 ± 2.66	14.92 ± 2.70	0.127*
CMT (μm)	393.98 ± 74.92	388.82 ± 69.65	399.23 ± 80.12	0.073*
gpRNFLT (μm)	105.66 ± 15.38	105.39 ± 17.41	105.88 ± 13.59	0.875*

Values are presented as n or mean ± standard deviation.

iERM idiopathic epiretinal membrane, *BSS* balanced salt solution, *Air* air tamponade, *IOL* intraocular lens, *VA* visual acuity, *logMAR* logarithm of the minimum angle of resolution, *AXL* axial length, *SE* spherical equivalent, *D* diopters, *IOP* intraocular pressure, *CMT* central macular thickness, *gpRNFLT* global peripapillary retinal nerve fiber layer thickness.

*p values were derived by independent t tests.

^†^*p* values were derived by Pearson’s chi-square tests.

### Visual outcomes and central macular thickness after epiretinal membrane removal with and without air tamponade

BCVA and IOP were not significantly different between the two groups for 2 years (Table 2). There was also no significant difference in macular thickness over the central, superior, nasal, inferior, or temporal regions between the two groups for 2 years (Table 3).

**Table 2:** Comparison of parameters of the BSS and air groups over 2 years.

Parameter	Group	Baseline	1 month	3 months	6 months	1 year	2 years	*p* value^†^
VA (logMAR)	BSS	0.36 ± 0.27	0.30 ± 0.22	0.21 ± 0.20	0.19 ± 0.18	0.17 ± 0.15	0.17 ± 0.14	< 0.001
	Air	0.36 ± 0.26	0.33 ± 0.23	0.25 ± 0.17	0.20 ± 0.15	0.18 ± 0.13	0.15 ± 0.11	< 0.001
	*p* value*	0.425	0.494	0.194	0.674	0.680	0.519	
SE	BSS	0.27 ± 1.99	− 0.50 ± 0.83	− 0.53 ± 0.77	− 0.48 ± 0.86	− 0.28 ± 0.81	− 0.41 ± 0.79	0.040
	Air	− 0.77 ± 3.15	− 0.76 ± 1.11	− 0.53 ± 0.75	− 0.57 ± 0.85	− 0.51 ± 0.89	− 0.52 ± 0.89	0.581
	*p* value*	0.203	0.167	0.962	0.551	0.164	0.603	
IOP	BSS	15.46 ± 3.04	14.62 ± 3.18	13.90 ± 2.77	14.29 ± 3.10	14.49 ± 3.13	13.94 ± 2.72	0.004
	Air	14.94 ± 2.49	14.46 ± 3.35	13.67 ± 2.82	14.26 ± 2.95	14.48 ± 2.80	14.30 ± 3.32	0.173
	*p* value*	0.127	0.794	0.655	0.967	0.986	0.624	

Values are presented as n or mean ± standard deviation.

*BSS* balanced salt solution, *Air* air tamponade, *VA* visual acuity, *logMAR* logarithm of the minimum angle of resolution, *SE* spherical equivalent, *IOP* intraocular pressure.

*p values were derived by independent t-tests.

^†^*p* values were derived by paired t-tests for the preoperative and second year postoperative values.

**Table 3:** Comparison of the macular thickness of the BSS and air groups over 2 years.

Parameter	Location	Central	Superior	Nasal	Inferior	Temporal
Preoperative	BSS	388.82 ± 69.65	331.08 ± 46.18	338.18 ± 35.76	307.61 ± 37.53	319.17 ± 42.34
	Air	399.23 ± 80.12	332.49 ± 40.30	348.74 ± 34.84	318.82 ± 47.60	320.71 ± 41.90
	*p* value	0.073	0.575	0.791	0.074	0.992
1 month	BSS	409.15 ± 67.80	332.62 ± 32.15	351.48 ± 27.17	312.69 ± 23.61	310.08 ± 33.41
	Air	393.14 ± 56.31	329.53 ± 28.08	358.16 ± 27.63	320.17 ± 26.98	304.69 ± 33.96
	*p* value	0.183	0.376	0.930	0.479	0.950
3 months	BSS	377.49 ± 46.10	315.04 ± 25.23	339.42 ± 25.32	302.21 ± 25.80	294.89 ± 25.55
	Air	381.41 ± 47.62	321.02 ± 23.05	346.89 ± 21.42	310.52 ± 26.75	302.15 ± 24.14
	*p* value	0.614	0.468	0.211	0.917	0.465
6 months	BSS	374.18 ± 46.41	309.93 ± 25.05	333.20 ± 22.76	295.06 ± 21.43	291.11 ± 24.40
	Air	370.15 ± 48.81	311.26 ± 25.55	337.75 ± 22.19	300.91 ± 24.94	293.87 ± 28.16
	*p* value	0.496	0.897	0.734	0.639	0.591
1 year	BSS	361.75 ± 38.12	300.13 ± 19.86	328.36 ± 24.41	289.69 ± 21.23	286.33 ± 23.97
	Air	363.51 ± 46.50	306.20 ± 24.63	335.37 ± 18.34	296.32 ± 25.29	290.34 ± 29.13
	*p* value	0.133	0.419	0.131	0.707	0.199
2 years	BSS	361.89 ± 43.49	303.42 ± 31.33	321.89 ± 22.25	286.22 ± 22.15	285.97 ± 25.52
	Air	355.32 ± 51.38	302.50 ± 29.36	330.64 ± 20.86	299.64 ± 43.62	289.96 ± 33.40
	*p* value	0.302	0.847	0.484	0.052	0.260

Values are presented as n or mean ± standard deviation.

*BSS* balanced salt solution; *Air* air tamponade.

*p* values were derived by independent t-tests.

### Temporal changes of best-corrected visual acuity and central macular thickness after epiretinal membrane removal with and without air tamponade

**Figure 1:**
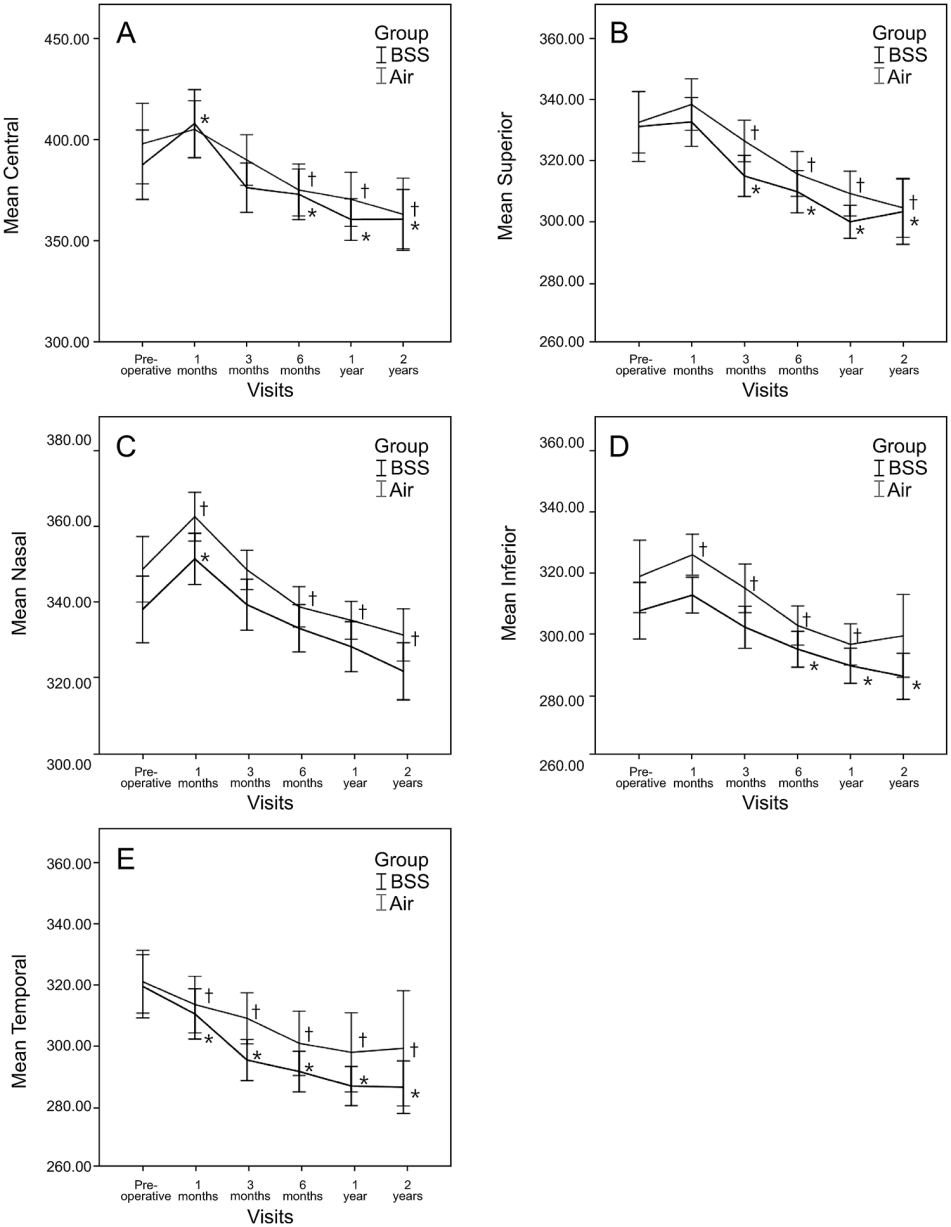
Changes in macular thickness over 2 years. (**A**) Mean central macular thickness. (**B**) Mean superior segment macular thickness. (**C**) Mean nasal segment macular thickness. (**D**) Mean inferior segment macular thickness. (**E**) Mean temporal segment macular thickness changes in the BSS and Air groups. There was no significant difference between the two groups for 2 years. Significant change compared to preoperative values in the BSS and air groups. Abbreviations: *BSS* balanced salt solution, *Air* air tamponade.

The preoperative and postoperative BCVA and CMT significantly differed. BCVA was improved from 0.36 ± 0.26 logMAR to 0.15 ± 0.11 logMAR in the air group (*p* < 0.001) and from 0.36 ± 0.27 logMAR to 0.17 ± 0.14 logMAR in the BSS group (*p* < 0.001) (Table 2). CMT was decreased from 399.23 ± 80.12 μm to 355.32 ± 51.38 μm in the air group (*p* < 0.001) and from 388.82 ± 69.65 μm to 361.89 ± 43.49 μm in the BSS group (*p* = 0.021) (Fig. 1).

### pRNFL thickness after epiretinal membrane removal with and without air tamponade

**Figure 2:**
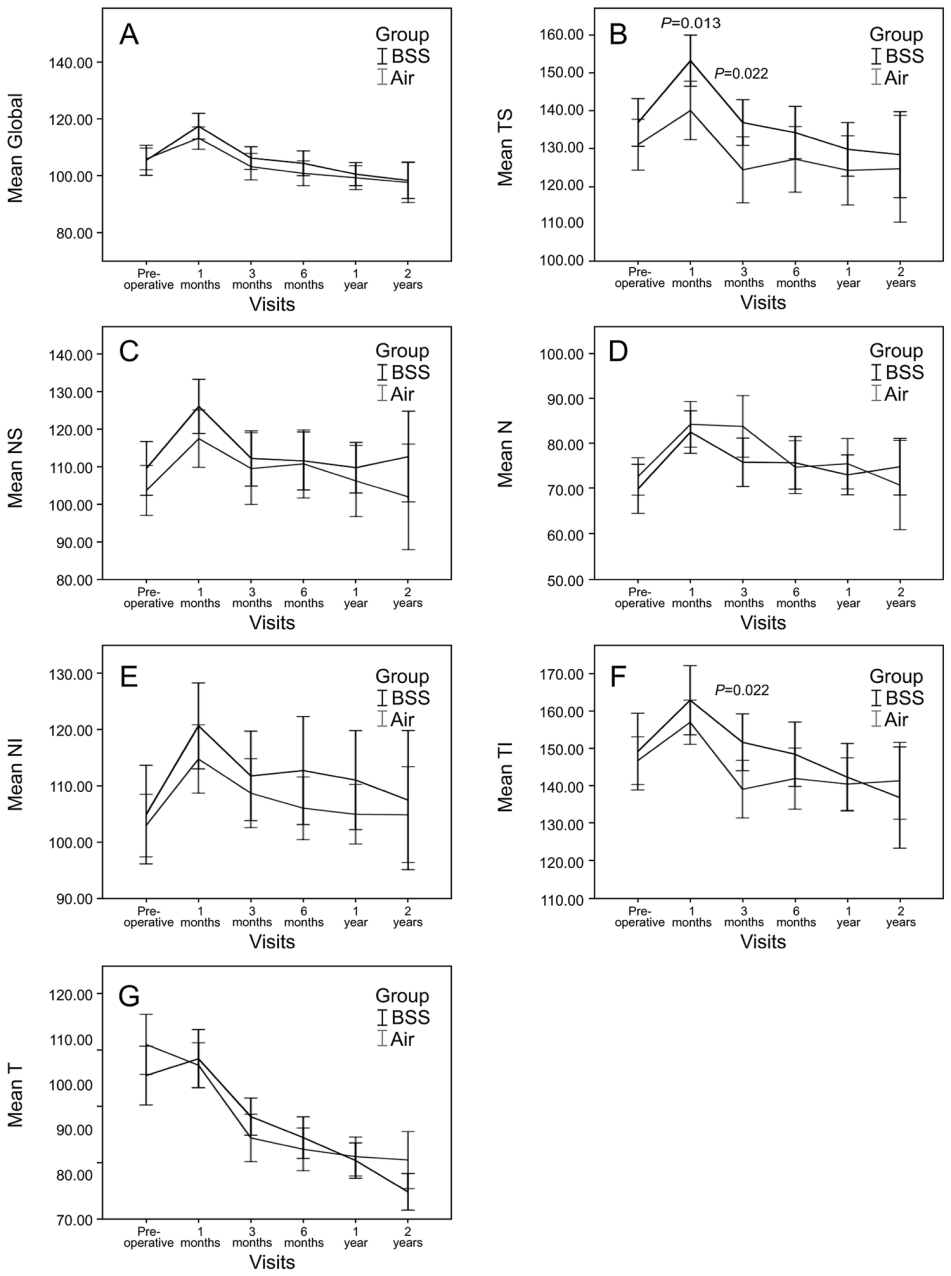
Comparison of peripapillary RNFL over 2 years between the BSS and air groups. (**A**) Global RNFL thickness. (**B**) Mean TS segment RNFL thickness. (**C**) Mean NS segment RNFL thickness. (**D**) Mean N segment RNFL thickness. (**E**) Mean NI segment RNFL thickness. (**F**) Mean TI segment RNFL thickness. (**G**) Mean T segment RNFL thickness. (**A**, C–**E**, **G**) There were no significant differences between the two groups for 2 years. (**B**) There were significant differences between the two groups at 1 and 3 months (*p* = 0*.*01 and *p* = 0*.*02, respectively). (**F**) There was a significant difference between the two groups at 3 months (*p* = 0*.*022). Abbreviations: *BSS* balanced salt solution, *Air* air tamponade, *RNFL* retinal nerve fiber layer, *TS* Temporal superior, *NS* superior nasal, *N* nasal, *NI* inferior nasal, *TI* inferior temporal, *T* temporal.

There was no significant difference in the global pRNFL at 2 years after surgery (Table 4; Fig. 2). However, there was a significant difference in the TS sector at the 1-month follow-up. The air group (139.89 ± 26.20 μm) had a significantly lower thickness than the BSS group (153.13 ± 21.28 μm) (*p* = 0.013). At 3 months after surgery, the air group had lower TI and TS sector values than the BSS group (*p* = 0.022, *p* = 0.022, respectively). There was no significant difference between the two groups over the entire sectors from 6 months to 2 years.

**Table 4:** Comparison of peripapillary RNFL thickness of the BSS and air groups over 2 years.

Parameter	Location	Global	TS	NS	N	NI	TI	T
Preop	BSS	105.39 ± 17.41	136.68 ± 20.93	109.48 ± 23.50	69.98 ± 17.88	104.86 ± 28.73	149.14 ± 33.55	101.73 ± 21.43
	Air	105.88 ± 13.59	130.79 ± 24.34	103.63 ± 23.84	72.71 ± 14.88	102.92 ± 19.90	146.73 ± 22.83	108.62 ± 23.82
	*p* value	0.875	0.211	0.232	0.415	0.698	0.678	0.143
1 month	BSS	117.40 ± 14.17	153.13 ± 21.28	125.98 ± 22.55	82.55 ± 14.68	120.60 ± 23.85	162.80 ± 28.69	105.45 ± 20.12
	Air	113.20 ± 13.24	139.89 ± 26.20	117.39 ± 25.60	84.26 ± 17.05	114.72 ± 20.38	156.93 ± 19.77	104.02 ± 16.70
	*p* value	0.159	0.013	0.105	0.622	0.221	0.268	0.720
3 months	BSS	106.17 ± 11.72	136.67 ± 18.04	112.14 ± 21.72	75.86 ± 15.83	111.72 ± 23.45	151.61 ± 22.29	92.61 ± 12.19
	Air	103.15 ± 14.36	124.10 ± 27.09	109.44 ± 29.55	83.79 ± 21.00	108.67 ± 18.79	139.15 ± 23.61	87.90 ± 16.22
	*p* value	0.325	0.022	0.655	0.070	0.534	0.022	0.162
6 months	BSS	104.32 ± 12.51	134.00 ± 20.01	111.47 ± 22.13	75.74 ± 16.69	112.68 ± 27.42	148.44 ± 24.53	87.97 ± 13.23
	Air	100.78 ± 13.69	126.90 ± 27.3	110.68 ± 28.28	74.78 ± 18.28	106.00 ± 17.35	141.98 ± 25.33	85.38 ± 14.78
	*p* value	0.252	0.213	0.895	0.815	0.208	0.271	0.432
1 year	BSS	100.50 ± 12.82	129.53 ± 22.32	109.68 ± 21.03	73.08 ± 13.81	110.98 ± 27.42	142.35 ± 28.05	82.88 ± 12.27
	Air	99.26 ± 13.75	123.95 ± 29.92	106.14 ± 30.78	75.53 ± 18.16	104.93 ± 17.16	140.53 ± 22.65	83.77 ± 13.98
	*p* value	0.672	0.342	0.546	0.492	0.229	0.746	0.759
2 years	BSS	98.31 ± 12.06	128.13 ± 21.47	112.63 ± 22.64	74.88 ± 11.76	107.44 ± 23.13	130.75 ± 40.06	75.94 ± 7.58
	Air	97.57 ± 15.55	124.38 ± 31.20	101.90 ± 30.85	70.81 ± 21.71	104.86 ± 18.66	141.38 ± 22.48	83.00 ± 13.92
	*p* value	0.876	0.684	0.250	0.504	0.709	0.312	0.076

Values are presented as n or mean ± standard deviation.

*Preop* preoperative, *RNFL* retinal nerve fiber layer, *BSS* balanced salt solution, *Air* air tamponade, *TS* temporal superior, *NS* superior nasal, *N* nasal, *NI* inferior nasal, *TI* inferior temporal, *T* temporal.

*p* values were derived by independent t-tests.

### Temporal changes in pRNFL thickness after epiretinal membrane removal with and without air tamponade

The pRNFL values in both the air and BSS groups significantly increased in all areas, except for the T sector of the air group at 1 month after surgery, and then gradually decreased at 1 year (Table 5). The thickness of the T and TS sectors of both groups showed a significant decrease at 1 year after surgery compared with that at baseline. The thickness of the TI sector of the air group also showed a significant decrease at 1 year after surgery compared with that at baseline, whereas the decrease in the TI sector of the BSS group was not significant (*p* = 0.086) at 1 year after surgery.

**Table 5:** Comparison of peripapillary RNFL thickness before and after surgery in the BSS and air groups.

Parameter	Location	Global	TS	NS	N	NI	TI	T
Preop	BSS	105.39 ± 17.41	136.68 ± 20.93	109.48 ± 23.50	69.98 ± 17.88	104.86 ± 28.73	149.14 ± 33.55	101.73 ± 21.43
	Air	105.88 ± 13.59	130.79 ± 24.34	103.63 ± 23.84	72.71 ± 14.88	102.92 ± 19.90	146.73 ± 22.83	108.62 ± 23.82
1 month	BSS	117.40 ± 14.17 (< 0.001)	153.13 ± 21.28 (< 0.001)	125.98 ± 22.55 (< 0.001)	82.55 ± 14.68 (< 0.001)	120.60 ± 23.85 (< 0.001)	162.80 ± 28.69 (0.001)	105.45 ± 20.12 (0.436)
	Air	113.20 ± 13.24 (< 0.001)	139.89 ± 26.20 (< 0.001)	117.39 ± 25.60 (< 0.001)	84.26 ± 17.05 (< 0.001)	114.72 ± 20.38 (< 0.001)	156.93 ± 19.77 (0.003)	104.02 ± 16.70 (0.131)
3 months	BSS	106.17 ± 11.72 (0.876)	136.67 ± 18.04 (0.692)	112.14 ± 21.72 (0.383)	75.86 ± 15.83 (< 0.001)	111.72 ± 23.45 (0.006)	151.61 ± 22.29 (0.339)	92.61 ± 12.19 (< 0.001)
	Air	103.15 ± 14.36 (0.254)	124.1 ± 27.09 (0.128)	109.44 ± 29.55 (0.013)	83.79 ± 21.00 (0.015)	108.67 ± 18.79 (0.028)	139.15 ± 23.61 (0.043)	87.90 ± 16.22 (< 0.001)
6 months	BSS	104.32 ± 12.51 (0.073)	134 ± 20.01 (0.111)	111.47 ± 22.13 (0.675)	75.74 ± 16.69 (0.051)	112.68 ± 27.42 (0.625)	148.44 ± 24.53 (0.171)	87.97 ± 13.23 (< 0.001)
	Air	100.78 ± 13.69 (0.002)	126.9 ± 27.30 (0.027)	110.68 ± 28.28 (0.103)	74.78 ± 18.28 (0.057)	106.00 ± 17.35 (0.248)	141.98 ± 25.33 (0.047)	85.38 ± 14.78 (< 0.001)
1 year	BSS	100.5 ± 12.82 (0.017)	129.53 ± 22.32 (0.029)	109.68 ± 21.03 (0.896)	73.08 ± 13.81 (0.310)	110.98 ± 27.42 (0.264)	142.35 ± 28.05 (0.086)	82.88 ± 12.27 (< 0.001)
	Air	99.26 ± 13.75 (< 0.001)	123.95 ± 29.92 (0.016)	106.14 ± 30.78 (0.456)	75.53 ± 18.16 (0.128)	104.93 ± 17.16 (0.442)	140.53 ± 22.65 (0.006)	83.77 ± 13.98 (< 0.001)
2 years	BSS	98.31 ± 12.06 (0.032)	128.13 ± 21.47 (0.149)	112.63 ± 22.64 (0.874)	74.88 ± 11.76 (0.291)	107.44 ± 23.13 (0.203)	130.75 ± 40.06 (0.07)	75.94 ± 7.58 (0.001)
	Air	97.57 ± 15.55 (0.005)	124.38 ± 31.20 (0.069)	101.9 ± 30.85 (0.906)	70.81 ± 21.71 (0.819)	104.86 ± 18.66 (0.968)	141.38 ± 22.48 (0.14)	83.00 ± 13.92 (< 0.001)

Values are presented as n or mean ± standard deviation.

*Preop* preoperative, *RNFL* retinal nerve fiber layer, *BSS* balanced salt solution, *Air* air tamponade, *TS* temporal superior, *NS* superior nasal, *N* nasal, *NI* inferior nasal, *TI* inferior temporal, *T* temporal.

The value in ( ) is the *p* value compared to before operation (paired t test).

At the end, in the second year after surgery, there was a significant difference only in the T sector of both groups. The T sector of the BSS group decreased from 101.73 ± 21.43 μm preoperatively to 75.94 ± 7.58 μm (*p* = 0.001), and that in the air group decreased from 108.62 ± 23.82 μm preoperatively to 83.00 ± 13.92 μm (*p* < 0.001). In both groups, the nasal side (N, NI, and NS sector) returned to its preoperative value within 6 months and did not show a significant difference when compared with the preoperative values.

### Baseline values affecting final global pRNFL thickness and final central macular thickness

Final global pRNFL thickness had a significant relationship with preoperative global pRNFL thickness, whereas other factors including age, sex, SE, IOP, AXL, and the presence or absence of air tamponade were not significantly related (Table 6). Final CMT showed a significant association with preoperative CMT, but lacked a significant relationship with other factors (Table 6).

**Table 6:** Values affecting final global peripapillary RNFL thickness and final CMT through multiple regression analysis.

Preoperative values	Final global peripapillary RNFL		Final CMT	
	Standardized coefficients	*p* value	Standardized coefficients	*p* value
Age	− .084	.657	.096	.492
SEX	.027	.859	− .017	.889
IOP	.005	.973	− .110	.387
SE	.143	.614	− .049	.785
AXL	− .037	.889	− .057	.749
Preoperative CMT	− .257	.124	**.669**	** < .001**
Preoperative gpRNFL	**.692**	** < 0.001**	.184	.161
Air tamponade^a^	.013	.934	.013	.915

Bold font indicates statistically significant values (*p* value < 0.05).

*SE* spherical equivalent, *IOP* intraocular pressure, *AXL* axial length, *CMT* central macular thickness, *RNFL* retinal nerve fiber layer, *gpRNFL* global peripapillary retinal nerve fiber layer.

^a^Intraoperative value that indicates the presence or absence of an air tamponade during surgery.

## Discussion

In this study, we compared the use of air tamponade and no tamponade after PPV surgery for iERM. We found that every parameter, BCVA, CMT, and pRNFL thickness, improved gradually throughout 2 years postoperatively in both groups. Moreover, there was no significant difference in each parameter assessed between the two groups. Previously, two studies compared the use of air and BSS tamponade after vitreoretinal surgery^18,19^. One of the studies compared the difference in visual acuity between the two groups after lamellar macular hole surgery^19^, and the other compared the visual acuity and foveal contour changes between the two groups after iERM surgery^18^. However, prior to this report, there was no study comparing the difference in visual acuity, macular thickness, and pRNFL thickness between these two groups after iERM surgery with a long-term follow-up (2 years). In particular, this is the first study to compare pRNFL between two groups after iERM surgery.

Postoperative visual acuity and CMT were not significantly different between the two groups, but pRNFL showed a temporary difference after the surgery. At 1 month after surgery, the air group showed lower thickness than the BSS group in the TS segment, and at 3 months, the air group showed lower thickness in the TI segment as well as in the TS segment. This difference could be attributed to the compression effect of air tamponade. However, it is noteworthy that the difference in the pRNFL thickness between the two groups was significant up to 3 months, even though air tamponade in the vitreous cavity generally lasts for less than 1 month. Although the pRNFL values were not compared previously, Leitritz et al.^18^ compared the visual acuity, foveal surface symmetry, area-matched thickness, and area-matched contour of the air and BSS groups after ERM surgery. In that study, visual acuity, area-matched thickness, and area-matched contour did not differ between the two groups, but the foveal surface symmetry, comparing the shape of the nasal side of the macula and the temporal side of the macula with the fovea, was significantly higher in the air group up to 3 months postoperatively. These results are also one of the reasons for the inference that the tamponade effect of air can last up to 3 months. However, they only analyzed changes for up to 3 months, whereas we analyzed results up to 2 years and found that no significant difference in the pRNFL between the two groups from 3 months to 2 years.

When comparing the preoperative to the postoperative 1-month pRNFL values for each group, the BSS group had an increase in thickness in all areas, while the air group had an increase in thickness in all areas with the exception of the temporal sector. For both groups, after the 1-month measurements, the thickness slowly decreased and by the 2-year time point, there was no significant difference from the preoperative values in all sectors, with the exception of the pRNFL thickness of the temporal sector that was significantly decreased. These observations are in line with previous studies. In the study by Balducci et al.^20^, 30 patients underwent vitrectomy, and the pRNFL thickness was increased in all areas, except the temporal sector, at 1 month; these then gradually returned to preoperative values. At 6 months, only the thickness of the temporal area was significantly reduced in their study. Lee et al.^21^ also reported a significant decrease in pRNFL thickness at the temporal sector in 31 patients with ERM at 3, 6, and 12 months postoperatively. In our study, the thickness of the nasal side (N, NI, and NS sector) was restored to the preoperative value within 6 months in both groups and did not show a significant change even 2 years after the surgery. Lee et al.^21^ also reported no change in the nasal side at 1, 3, 6 and 12 months after surgery. These results may be due to the fact that the nasal side was hardly affected by ERM, and the effect of air tamponade also did not seem to have a significant effect on the nasal side of pRNFL.

A transient increase in the thickness of the pRNFL, which occurs 1 month after such surgery, may originate from the inflammatory response caused by the vitrectomy surgery itself. Additionally, according to the previous reports, direct mechanical damage to the optic head by the procedure such as ILM peeling, or inducing PVD with ocutome, or retinal toxicity due to ICG dye could be the causes^17,20,22,23^. The thickness of the temporal area gradually decreases, which may be due to the resolution of the traction force generated by ERM after surgery^21^ or due to damage to the inner retinal layer during ILM peeling^20^.

Visual acuity improved significantly in both groups postoperatively. Leitritz et al.^18^ reported no difference in visual acuity between the two groups during the short (3 month) follow-up period after vitrectomy due to iERM. From a practitioner’s point of view, tamponade in the eye may cause lower vision and slower vision recovery immediately after surgery compared to no tamponade. However, our results with air tamponade showed that the visual acuity was identical between the two groups at 1 month postoperatively. In this study, there was also no significant difference in visual acuity between the two groups at all time points during the 2 years of follow-up.

This study has some limitations. First, the lack of randomization makes it entirely possible that there are baseline differences in disease severity between the two groups. Second, we enrolled a relatively small number of patients. To overcome this limitation, we refined our study design: we performed phacoemulsification and intraocular lens insertion on all 132 phakic eyes to exclude the effect of medial opacity on visual acuity according to the time change; the ILM peeling on every patient was performed by a single surgeon using the same technique; we excluded all patients with outpatient clinic or intraoperative finding of no PVD to remove or minimize the effect of PVD induction procedure on the pRNFL. Finally, we did not perform a visual field test. If we performed visual field examination, we would be able to obtain more information on how the change in pRNFL affects the visual field. Despite these limitations, this study is meaningful because of the long-term follow-up of 2 years, and it is the first report to prospectively compare visual acuity, macular thickness, and pRNFL between an air tamponade group and a no tamponade group in iERM surgery.

In conclusion, our study showed that there was no difference in postoperative visual acuity, macular thickness, and pRNFL thickness when air tamponade versus non-tamponade was performed during iERM surgery. This demonstrates that air tamponade might not be an imperative procedure for iERM surgery and has no additional benefit.

## Methods

### Subjects

This study was a prospective cohort study. Enrolled patients underwent surgery at Hangil Eye Hospital between January 2015 and January 2018. The study included only patients with confirmed iERM found using the Spectralis domain optical coherence tomography system (SD-OCT, Spectralis Version 5.6.1.0, Heidelberg Engineering, Heidelberg, Germany). Patients with other diseases that might affect pRNFL thickness and macular thickness, such as glaucoma, diabetic retinopathy, retinal vein occlusion, and age-related macular degeneration were excluded. Additionally, patients that did not present with posterior vitreous detachment (PVD) either intraoperatively, or in the outpatient clinic were excluded from the study.

### Surgical methods

Only one surgeon (DDH) performed all the surgeries. A 3-port 25-gauge scleral tunnel transconjunctival sutureless vitrectomy was performed using the Constellation Vision Surgical System (Alcon Surgical, Fort Worth, TX, USA). ERM was removed under 2% lidocaine sub-Tenon anesthesia with monitored anesthesia care. Cataract surgery was also performed with vitrectomy in all phakic patients. After core and peripheral vitrectomy were performed, epiretinal membrane removal and ILM peeling were carefully performed with retinal forceps. Triamcinolone agent (MaQaid, Wakamoto Pharmaceutical Co., Ltd., Tokyo, Japan) was used for membrane peeling in some cases. If either an atrophic hole or lattice degeneration was present, air endo-tamponade was performed. The air tamponade was a full fill. These patients were then recommended to maintain a prone position for 1-day post operation. The surgery was completed after confirmation of the absence of any remaining membrane and macular hole using the intraoperative OCT (RESCAN 700, Zeiss, Germany).

### Ophthalmic examinations

Ophthalmologic examinations including slit-lamp examination, fundoscopy, best-corrected visual acuity (BCVA), intraocular pressure (IOP), manifest refraction, central macular thickness (CMT), and pRNFL thickness were measured at the baseline visit and at 1 month, 3 months, 6 months, 1 year, and 2 years after surgery. CMT and pRNFL thickness were measured by SD-OCT, using the equipped software. pRNFL thickness was measured in six sectors: temporal (T, 315°–45°), superior temporal (TS, 45°–90°), superior nasal (NS, 90°–135°), nasal (N, 135°–225°), inferior nasal (NI, 225°–270°), and inferior temporal (TI, 270°–315°), together with the papillomacular bundle (338°–8°; Spectralis Nsite Axonal Analytics Software; Heidelberg Engineering) thickness. The global pRNFL thickness was obtained by averaging the total 360° pRNFL thicknesses. The thicknesses in the central and outer four quadrants were measured (scanning area, 6 × 6 mm, centered at the fovea), and the CMT was obtained from the Early Treatment Diabetic Retinopathy Study circle on the macula.

### Ethics statement

This study was approved by the Institutional Review Board of Hangil Eye Hospital and was carried out in accordance with the tenets of the Declaration of Helsinki; informed consent was obtained from all the patients.

### Statistical analysis

Statistical analyses were performed using a commercially available software package (SPSS Statistics version 23; IBM Corp., Armonk, NY, USA). Independent t-test and Pearson’s chi-square test were performed to compare the characteristics of the two patient groups: air tamponade (air group) and balanced salt solution (no tamponade; BSS group). Paired t-tests were used to compare pRNFL thickness before and after surgery in the two groups. A multiple regression model was used to evaluate whether there were preoperative factors affecting final global pRNFL thickness and final CMT. Age, sex, SE, IOP, AXL, the presence or absence of air tamponade, preoperative global pRNFL and preoperative CMT values were corrected. *p* values < 0.05 were considered significant.
